# Crystallization Kinetics Analysis of the Amorphouse Mg_72_Zn_24_Ca_4_ Alloy at the Isothermal Annealing Temperature of 507 K

**DOI:** 10.3390/ma13122815

**Published:** 2020-06-23

**Authors:** Janusz Lelito

**Affiliations:** Faculty of Foundry Engineering, AGH University of Science and Technology, Al. A. Mickiewicza 30, 30-059 Kraków, Poland; lelito@agh.edu.pl

**Keywords:** Mg_72_Zn_24_Ca_4_ alloy, metallic glass, crystallization, kinetic model of Kolmogorov-Johnson-Mehl-Avrami-Evans

## Abstract

This paper presents tests of metallic glass based on Mg_72_Zn_24_Ca_4_ alloy. Metallic glass was made using induction melting and further injection on a rotating copper wheel. A differential scanning calorimeter (DSC) was used to investigate the phase transformation of an amorphous ribbon. The tests were carried out at an isothermal annealing temperature of 507 K. The Kolmogorov-Johnson-Mahl-Avrami-Evans model was used to analyze the crystallization kinetics of the amorphous Mg_72_Zn_24_Ca_4_ alloy. In this model, both Avrami’s exponent *n* and transformation rate constant K were analyzed. Both of these kinetic parameters were examined as a function of time and the solid fraction. The Avrami exponent *n* value at the beginning of the crystallization process has value *n* = 1.9 and at the end of the crystallization process has value *n* = 3.6. The kinetic constant *K* values change in the opposite way as the exponent *n*. At the beginning of the crystallization process the constant *K* has value *K* = 9.19 × 10^−7^ s^−n^ (ln(*K*) = −13.9) and at the end of the crystallization process has the value *K* = 6.19 × 10^−9^ s^−n^ (ln(*K*) = −18.9). These parameters behave similarly, analyzing them as a function of the duration of the isothermal transformation. The exponent *n* increases and the constant *K* decreases with the duration of the crystallization process. With such a change of the Avrami exponent *n* and the transformation rate constant *K*, the crystallization process is controlled by the 3D growth on predetermined nuclei. Because each metallic glass has a place for heterogeneous nucleation, so called pre-existing nuclei, in which nucleation is strengthened and the energy barrier is lowered. These nuclei along with possible surface-induced crystallization, lead to rapid nucleation at the beginning of the process, and therefore a larger transformed fraction than expected for purely uniform nucleation. These sites are used and saturated with time, followed mainly by homogeneous nucleation. In addition, such a high value of the Avrami exponent *n* at the end of the crystallization process can cause the impingement effect, heterogeneous distribution of nuclei and the diffusion-controlled grain growth in the Mg_72_Zn_24_Ca_4_ metallic glassy alloy.

## 1. Introduction

Magnesium alloys are characterized by low density and high mechanical properties, thanks to which they have found application in many industries, primarily in aviation and the automotive industry [1,2,3,4]. In addition to these advantages, magnesium alloys also show good biocompatibility with the human body. This feature speaks for the use of magnesium alloys also in medicine. Biodegradable magnesium implants after tissue healing would dissolve themselves and then be excreted or absorbed by the human body [5,6]. This would mean shortening the recovery of patients, as well as reducing the cost of treatment. However, the excessive degradation rate and hydrogen evolution [7,8] led to the abandonment of further work on the biomedical use of crystalline magnesium alloy.

Recent years have brought increasing interest in magnesium and its alloys as a material for biodegradable metal for orthopedic implants. Studies have shown that magnesium alloys can be used, but in the form of amorphous metal glasses containing magnesium (Mg), zinc (Zn) and calcium (Ca) [9], which are also present in the human body. The amorphous nature of metallic glasses can provide a material with higher corrosion resistance and better mechanical properties than crystalline materials. However, the widespread use of amorphous alloys is limited due to their low plasticity [10]. Therefore, the partial crystallization process of amorphous alloys can be a solution that allows improving plastic properties with a slight deterioration of corrosion properties. Therefore, it is important to understand the kinetics of crystallization of metallic glasses based on Mg. 

The Mg_72_Zn_24_Ca_4_ alloy was selected for this study because its chemical composition is close to the chemical composition of the eutectic point for MgZnCa alloy. It is obvious that the alloys which have the chemical composition close to the eutectic point can be easily transformed to the metallic glasses. The transformation of alloys to the metallic glasses is related with the cooling rate of liquid alloys. The alloys which have significantly different chemical composition compared to the chemical composition of the eutectic point, require a higher cooling rate.

The crystallization rate in metallic glasses depends on the rate of nucleation and the rate of crystal growth. The kinetic model developed independently in 1930 and 1940 by Kolmogorov [11], Johnson and Mehl [12], Avrami [13,14,15] and Evans [16] is used to determine the crystallization rate. In the case where the nucleation rate (*J*) and growth rate (*u*) [17] are independent of time and the reaction is controlled by the interface, then the kinetics of the formation of spherical crystals in glassy alloys can be described by the general exponential equation for the phase transformed fraction *X*:

(1) $$X\left(t\right)=1-\exp\left(-\frac{\pi }{3}Ju^{3}t^{n}\right),$$

where *J* is nucleation rate, *u* is growth rate, *t* is time and *n* is the Avrami exponent. The value of this exponent should be close to 4 in the process of nucleation and three-dimensional growth. Such reactions are polymorphic and eutectic transformations. In the case of three-dimensional growth controlled by the interface, the kinetic constant *K* is equal to π/3 *Ju*3. In experimental studies, the Kolmogorov-Johnson-Mehl-Avrami-Evans equation (KJMAE) is often used to determine the transformation rate under isothermal conditions:

(2) $$X\left(t\right)=1-\exp\left(-Kt^{n}\right),$$

where: *X*—the solid fraction, and *K*—the transformation rate constant, depend on the nucleation rate and crystal growth rate; *n* — the Avrami exponent, is related to growth geometry and the nucleation mechanism. The exponent *n* should assume integer values, e.g., for three-dimensional growth and sporadic nucleation *n* = 3 + 1. In a situation where the transformation consists only in three-dimensional growth, then for the nucleus at the moment *t* = 0 (growth on predetermined nuclei), *n* = 3. In the case where the Avrami exponent *n* = 2.5, the transformation occurs at a constant nucleation rate and the growth is controlled by diffusion, whereas when *n* = 1.5 the transformation is based only on the growth controlled by diffusion.

The modification of the KJMAE equation, taking into account the incubation time *τ*, associated with the delay of the onset of transformation, takes the form:

(3) $$X\left(t\right)=1-\exp\left(-K\cdot {\left(t-\tau \right)}^{n}\right),$$

where: *τ* is the incubation time. The use of the KJMAE equation in the analysis of isothermal crystallization kinetics assumes the stationarity of the process, i.e. independence from the time of parameters *K* and *n*, and thus the rate of nucleation and growth. To determine the above quantities, the double-logarithmized form of Equation (3) is used:

(4) ln(−ln(1−X(t)))=lnK+nln(t−τ).

The values of *n* and *K* are determined from the slope and abscissa of the graph in coordinates, respectively ln(−ln(1 − *X*(*t*))) from ln(*t* − *τ*). In fact, the shape of such relationships is rarely linear, especially at the beginning and end of the process. The above non-linearity was observed for isothermal crystallization of deformed copper [18] as well as in a number of metallic glasses e.g., [19,20]. The use of the KJMAE equation, Equations (3) and (4), in the analysis of experimental kinetics of isothermal transformations leads to the determination of the type of nucleation mechanism and growth geometry determined by the exponent *n*.

## 2. Research Methodology

The experimental procedure and phase analysis ware described in the paper “Phase Transformation Analysis of the Amorphous Mg_72_Zn_24_Ca_4_ Alloy” published in Archives of Foundry Engineering [21]. In this paper the degree of metallic glass transformation was examined. The method of measuring the thermal effects of the amorphous alloy transformation into crystalline alloy was used. Therefore magnesium, zinc and calcium with a purity of 99.9% were used to obtain the Mg_72_Zn_24_Ca_4_ alloy. Then, the melting was carried out in a resistance furnace under argon as the inert gas. After melting the liquid alloy, it was cast into the steel mould to obtain a cylindrical sample. Then, the sample was melted again and cast using the melt spinning technique under argon as the inert gas. Such procedure allowed obtaining ribbon about 150 μm thick with an amorphous structure. X-ray diffraction was employed for identification of the amorphous nature of such-prepared ribbon. The crystallization kinetics of the amorphous alloy was investigated by continuous heating and isothermal annealing in a DSC Q20 (where DSC = differential scanning calorimeter, TA Instrument, Eschborn, Germany) under flow of high purity argon. In the case of continuous heating, the heating rate was 5 K/min. This allowed identification of the crystallization onset temperature (T = 524 K) and the melting onset temperature (T = 631 K). Values of these temperatures allowed predicting two isothermal annealing temperatures, 507 and 510 K, respectively. These temperatures are lower than the crystallization onset temperature. For the isothermal annealing, after the sample was heated at a rate of 80 K/min to a desired temperature (507 and 510 K). Heat release during isothermal annealing is probably associated with the nucleation process and the magnesium phase growth. On the basis of the research the following conclusions are made, that the heating the ribbons at the selected isothermal annealing temperature leads to intense heat production. The average rate of heat release during nucleation and the growth of the magnesium phase increases with the increase in the temperature of isothermal annealing in the range from 0.46 W/g for T = 507 K to 0.72 W/g for T = 510 K. This phenomenon is caused by an increase in the rate of zinc diffusion in the magnesium matrix at higher isothermal annealing temperatures. The increase in the temperature of isothermal annealing also reduces the duration of phase transformation from 360 s for T = 507 K to 228 s for T = 510 K. Isothermal annealing temperature has a similar effect on incubation time, causing its shortening from 300 s for T = 507 K to 252 s for T = 510 K.

The crystallization kinetics of the amorphous alloy was investigated by isothermal annealing in a TA DSC Q20 under flow of high purity argon. The measuring sensitivity of the TA DSC Q20 is 1 μW. The research of the phase transformation process was conducted under isothermal conditions for a temperature of 507 K. In the case of calorimetric methods, the determination of the phase transformation degree *X* was based on the measurement of the heat flux accompanying the transformation, *dH*/*dt*, using the equation:

(5) $$X\left(t\right)=\frac{\int_{\tau }^{t}\left(\frac{dH}{dt}\right)dt}{m\Delta H^{0}}$$

where *t* is the time, *τ* is the start of transformation time (incubation time), *m* is the sample mass and Δ*H*^0^ is the value of equilibrium enthalpy of transformation. The analysis of phase transformation kinetics was based on the designed relationship of the phase transformation degree dependence on time *X*(*t*) (isothermal conditions). The KJMAE model described by Equations (3) and (4) was used to analyze the phase transformation kinetics. These equations allow determining the constant transformation rate *K* and Avrami exponent *n*. The constant transformation rate *K* was calculated using the equation:

(6) $$K=\frac{-ln\left(1-X\right)}{{\left(t-\tau \right)}^{n}},$$

while the Avrami exponent *n* was calculated from the formula:

(7) $$n=\frac{\Delta \ln\left[-\ln\left(1-X\right)\right]}{\Delta \ln\left(t-\tau \right)}.$$

## 3. Results

**Figure 1:**
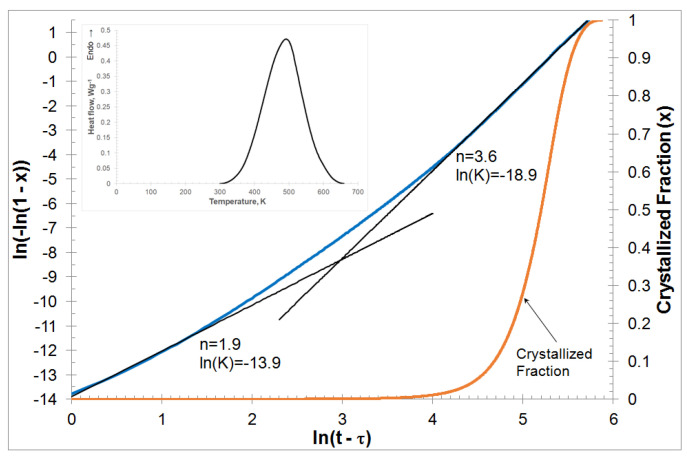
Isothermal differential scanning calorimeter (DSC) curve traces at annealing temperature equal to 507 K and the Kolmogorov-Johnsona-Mehla-Avrami-Evans plot and crystallized fraction for the isothermal crystallization process of Mg_72_Zn_24_Ca_4_ ribbon at annealing temperatures equal to 507 K, data obtained at temperatures above *T*_g_ as indicated. The values of n and ln(*K*) are also shown for two regions: early and late stages of crystallization.

Calorimetric studies of the phase transformation from amorphous to crystalline conditions under isothermal conditions consisted of heating the sample containing the amorphous phase initially to a temperature between the glass transition temperature and the crystallization temperature and registration of the heat flux while holding the sample at a constant temperature. The measurement was carried out at a temperature of 507 K. The phase transformation was visible as an endothermic peak indication as in [22]. The DSC calorimetric curve is shown in Figure 1. The analysis of the phase transformation kinetics of Mg_72_Zn_24_Ca_4_ metallic glass was based on the relationship of the phase transformation degree dependence on time, *X*(*t*), for isothermal conditions determined by integration of the DSC peak, Equation (5).

The crystalized fraction calculated from the curve of isothermal calorimetry and plotting KJMA ln(−ln(1 − *X*)) against ln(*t* − *τ*) is shown in Figure 1. The KJMAE plot is not linear in hundred percent, that is, the kinetic parameters *K* and *n* are dependent on time. When examining the crystallization of metallic glasses, the steady state of the process is usually taken into account, which usually corresponds to the volume fraction of the transformed phase in the range of 0.2–0.8; at which range the value of the Avrami exponent *n* is relatively constant. As can be seen, this is not true in this case. Probably, this may be due to the change in nucleation and growth rate over time.

Using linear approximation for the whole range of KJMAE relationships presented in Figure 1, the average values of ln(*K*) and *n* were determined, which are ln(*K*) = −16.7 and *n* = 3.1, respectively. The average value of the Avrami exponent *n* assumes a value close to 3. Assuming three-dimensional crystal growth, this indicates a predetermined nucleation mechanism.

Additionally, the above parameters were calculated for two crystallization ranges, i.e., the beginning and the end of the phase transformation. The values of these parameters are also shown in Figure 1. An increase in the Avrami exponent *n* with an increase in the volume fraction of the crystalline phase from *n* = 1.9 to *n* = 3.6 is visible. The kinetic constant *K* values change in the opposite way from *K* = 9.19 × 10^−7^ s^−n^ (ln(*K*) = −13.9) to *K* = 6.19 × 10^−9^ s^−n^ (ln(*K*) = −18.9).

**Figure 2:**
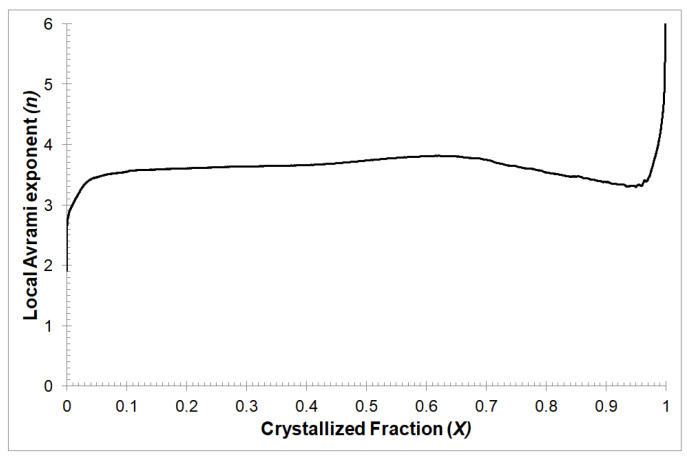
The local Avrami exponent *n*(*X*) varied with crystallized fraction *X* for isothermal annealing temperature equal to 507 K.

The local Avrami exponent *n*(*X*) value can be used for characterization of the nucleation process and the growth of precipitates during the isothermal annealing in dependence of the crystallized volumetric fraction *X*. The change of the Avrami exponent as a function of the volumetric fraction of the crystallized phase, is presented in Figure 2. It can be observed that, within the crystallized fraction range 0.1 < *X* < 0.8, the local Avrami exponent value is about 3.5. Such a value of the Avrami exponent *n* can cause 3D growth and sporadic nucleation. At the beginning and end of the crystallization process, the value of the Avrami exponent *n* decreases to about 3.0. Such an exponent value *n* can cause that crystallization process controlled by 3D growth on predetermined nuclei. In addition, such a high value of the Avrami exponent *n* at the end of the crystallization process can cause the impingement effect [23], heterogeneous distribution of nuclei [24,25] and the diffusion-controlled grain growth [26] in the Mg_72_Zn_24_Ca_4_ metallic glassy alloy.

**Figure 3:**
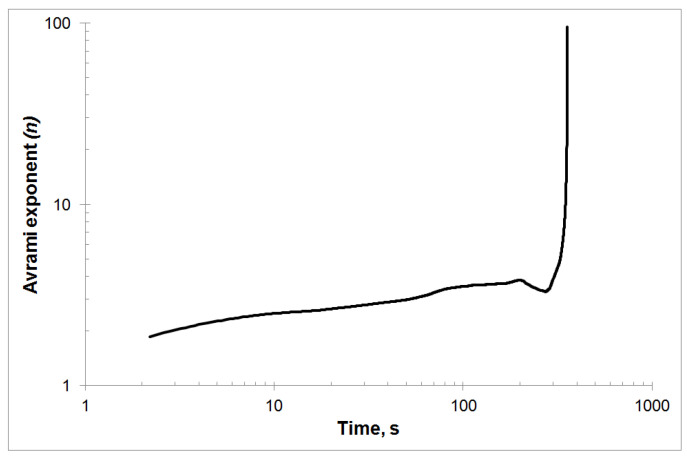
The Avrami exponent *n*(*t*) varied with time *t* for isothermal annealing temperature equal to 507 K.

The change of the Avrami exponent *n* over time is shown in Figure 3. As can be seen, the time dependence of the Avrami exponent *n* shows an increase in this parameter throughout the crystallization process. Only at the end of the crystallization process is a decrease in the value of the *n* parameter followed by an increase. Such a value of the Avrami exponent *n* may be associated with an amorphous phase loss at the end of the crystallization process.

**Figure 4:**
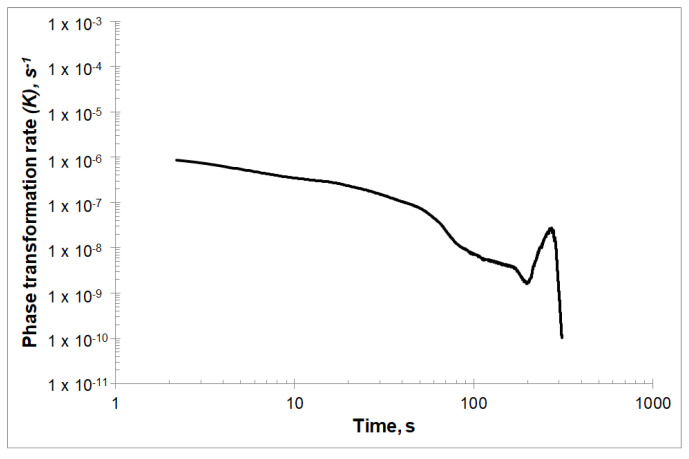
The phase transformation rate *K* varied with time *t* for isothermal annealing temperature equal to 507 K.

The results of the phase transformation rate *K*(*t*) are shown in Figure 4. The dependence of the phase transformation rate on the time *K*(*t*) for the first about 60 s decreases linearly, then rapidly decreases to a certain level and again decreases linearly for about the next 120 s. After this time, the phase transformation rate decreases and then rapidly increases, to achieve the value *K* = 1.8 × 10^−7^ s^−n^ after about 270 s of the crystallization process. From that moment, only a decrease in the phase transformation rate is observed.

The deviation of the crystallization rate *K* from the steady state conditions may result from the time changes of the Avrami exponent *n*. The deviations of the *K* rate at the beginning of the transformation may be related to the complex course of nucleation, while the decrease in the *K* rate in the final phase of transformation may result from the depletion of the amorphous phase.

**Figure 5:**
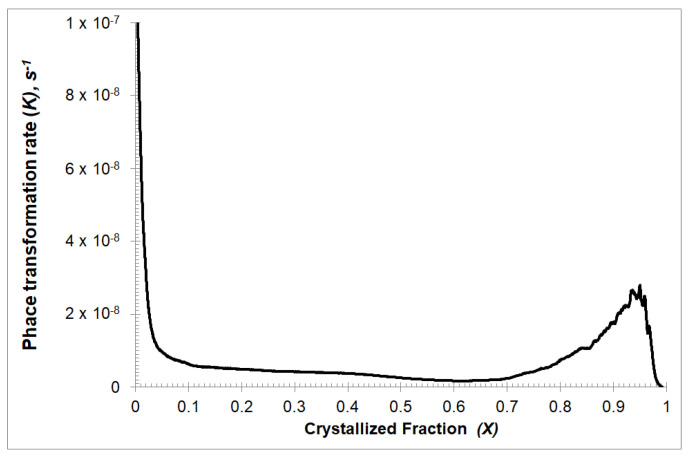
The phase transformation rate *K*(*X*) varied with crystallized fraction *X* for isothermal annealing temperature equal to 507 K.

The change of the phase transformation rate *K* as a function of the crystalline phase fraction is shown in Figure 5. It shows that the phase transformation rate remains constant around 10^−8^ in the solidified fraction range from 0.1 to 0.7. The end of the crystallization process can be divided into two stages. The first stage, i.e., from 0.7 to 0.95 of the solidified phase fraction, during which a sharp increase of the phase transformation rate is visible to about 3 × 10^−8^ and the second stage, during which the rapid decrease of the phase transformation rate occurs. The change of the phase transformation rate occurring during these last two stages of the crystallization process can be explained by the decreasing amount of amorphous phase between already touching grains. Then the dominating process is growth speed.

Analyzing the chart in Figure 5, a very high value of the phase transformation rate *K* is visible at the very beginning of the crystallization process. A higher value of *K* leads to a higher value of the nucleation rate *J*. This behavior is a good illustration of the fact that each metallic glass has heterogeneous nucleation sites where the nucleation is reinforced and the energy barrier lowered.

Average values of the conversion rate constant *K* = 5.6 × 10^−8^ s^−n^ and the Avrami exponent *n* = 3.1 were obtained. These values suggest that the crystallization process is controlled by three-dimensional growth on the nucleus. However, analyzing the course of the curve presented in Figure 1, there is a change in the value of the constant transformation rate from *K* = 9.2 × 10^−7^ s^−n^ to *K* = 6.2 × 10^−9^ s^−n^ at the beginning and end of the crystallization process, respectively. The value of the Avrami exponent also changed from *n* = 1.9 to *n* = 3.6. The Avrami exponent is lower in the initial crystallization step when the transformed fraction is less than 20% and then reaches its maximum in the last crystallization step. The maximum value of the Avrami exponent, during crystallization of the Mg_72_Zn_24_Ca_4_ amorphous alloy, has an exponent value *n* lower than 4. The glassy alloy Mg_72_Zn_24_Ca_4_ has a small proportion of the crystalline phase before crystallization, therefore this effect may to some extent be responsible for the relatively low value of the Avrami exponent of 3.6. In the case of oxide glasses, the nucleation rate *J* is lower at the initial stage of the process and reaches its maximum value in the steady state. In this work, the kinetic constant of the *K* transformation rate is higher at the initial stage of crystallization compared to the later stage of crystallization (Figure 1). In alloys showing eutectic transformation, in the absence of mutual interaction of grains, the rate of grain growth should be the same. Therefore, a higher *K* value should lead to a higher *J* value. This behavior can be a good illustration of the fact that all metallic glass has heterogeneous nucleation sites where the nucleation is reinforced and the energy barrier lowered.

These heterogeneous nucleation sites may be, e.g., pre-existing nuclei or cracks. These sites lead to rapid nucleation at the beginning of the process, and therefore increase the proportion of the transformed phase fraction more than would be expected for homogeneous nucleation. Also, different places for heterogeneous nucleation may show a different tendency to reduce the nucleation energy barrier expressed as the contact angle of the nucleus and catalyst [27,28], and thus affect the value of the exponent *n*.

Another factor responsible for the lower value of *n* is surface-induced crystallization, which leads to growth with lower dimensions compared to the growth with three dimensions occurring in the sample. Surface-induced crystallization often occurs in glassy samples in their initial state, which was observed by TEM [29,30] atomic force [31] and scanning tunneling microscopy [32]. This effect may also be responsible for lowering the energy barrier between the glassy phase and the crystalline phase. In addition, D.V. Louzguine-Luzgin et al. [33], studying the crystallization of the amorphous CuZrAlAg alloy, noticed no significant difference in the crystallization kinetics of volume and ribbon glassy samples. This phenomenon may indicate that it is not surface crystallization, but heterogeneous nucleation that plays the most important role, promoting faster crystallization at the initial stage. It should also be mentioned that heterogeneous nucleation may also occur at the initial stage of crystallization in nanostructured metallic glasses [34].

## 4. Conclusions

The kinetic constant of the phase transformation rate *K* in the glassy metallic alloy presented in this paper is higher at the first crystallization stage than at the second crystallization stage (Figure 1). The Avrami exponent *n* behaves quite differently than the kinetic constant *K*. The Avrami exponent *n* has a higher value in the second stage than in the first crystallization stage (Figure 1). Therefore, the crystallization process is controlled by the 3D growth on predetermined nuclei. This phenomenon can be explained by the fact that each metallic glass has a place for heterogeneous nucleation, in which nucleation is strengthened and the energy barrier is lowered. These sites are pre-existing nuclei that, along with possible surface-induced crystallization, can lead to rapid nucleation at the beginning of the process, and therefore a larger transformed fraction than expected for purely uniform nucleation. These sites are used and saturated with time, followed mainly by homogeneous nucleation. In addition, it should be mentioned that the amorphous alloy at the beginning of isothermal annealing already had a small amount of crystalline phase, which in addition could increase the concentration of the solidified volume through growth.

The crystallization analysis of the amorphous Mg_72_Zn_24_Ca_4_ alloy requires further research at additional annealing temperatures.
